# A nanoplex PCR assay for the rapid detection of vancomycin and bifunctional aminoglycoside resistance genes in *Enterococcus *species

**DOI:** 10.1186/1471-2180-7-112

**Published:** 2007-12-11

**Authors:** Chan Yean Yean, Lee Su Yin, Pattabhiraman Lalitha, Manickam Ravichandran

**Affiliations:** 1 Department of Medical Microbiology and Parasitology, School of Medical Sciences, Universiti Sains Malaysia, Malaysia; 2 School of Health Sciences, Universiti Sains Malaysia, Malaysia

## Abstract

**Background:**

Enterococci have emerged as a significant cause of nosocomial infections in many parts of the world over the last decade. The most common enterococci strains present in clinical isolates are *E. faecalis *and *E. faecium *which have acquired resistant to either gentamicin or vancomycin. The conventional culture test takes 2–5 days to yield complete information of the organism and its antibiotic sensitivity pattern. Hence our present study was focused on developing a nanoplex PCR assay for the rapid detection of vancomycin and bifunctional aminoglycoside resistant enterococci (V-BiA-RE). This assay simultaneously detects 8 genes namely 16S rRNA of *Enterococcus *genus, *ddl *of *E. faecalis *and *E. faecium*, *aac*A-*aph*D that encodes high level gentamicin resistance (HLGR), multilevel vancomycin resistant genotypes such as *van*A, *van*B, *van*C and *van*D and one internal control gene.

**Results:**

Unique and specific primer pairs were designed to amplify the 8 genes. The specificity of the primers was confirmed by DNA sequencing of the nanoplex PCR products and BLAST analysis. The sensitivity and specificity of V-BiA-RE nanoplex PCR assay was evaluated against the conventional culture method. The analytical sensitivity of the assay was found to be 1 ng at the DNA level while the analytical specificity was evaluated with 43 reference enterococci and non-enterococcal strains and was found to be 100%. The diagnostic accuracy was determined using 159 clinical specimens, which showed that 97% of the clinical isolates belonged to *E. faecalis*, of which 26% showed the HLGR genotype, but none were vancomycin resistant. The presence of an internal control in the V-BiA-RE nanoplex PCR assay helped us to rule out false negative cases.

**Conclusion:**

The nanoplex PCR assay is robust and can give results within 4 hours about the 8 genes that are essential for the identification of the most common *Enterococcus *spp. and their antibiotic sensitivity pattern. The PCR assay developed in this study can be used as an effective surveillance tool to study the prevalence of enterococci and their antibiotic resistance pattern in hospitals and farm animals.

## Background

Enterococci have emerged as prominent nosocomial pathogens that cause a variety of clinical infections. *Enterococcus faecalis *and *Enterococcus faecium *are clinically significant species that are implicated in 90% and 5–10% of enterococcal infections, respectively [1].

The widespread use and misuse of antimicrobials such as glycopeptides and aminoglycosides in human and livestock have resulted in the rapid increase of vancomycin and high level gentamicin-resistance in *Enterococcus *strains [2]. Vancomycin-resistant enterococci (VREs) were first isolated from patients in 1988 in the United Kingdom and France [3]. Since then, VRE have spread to many other countries including Malaysia, in the meat from livestock and by humans [4-7].

Vancomycin resistance in Enterococci have been classified based on the gene sequence and resistance characteristics [8]. The *van*A-type strains are resistant to high levels of both vancomycin and teicoplanin antimicrobials (MIC ≥ 64 μg/ml and >16 μg/ml, respectively). *van*B-type strains are resistant to a wide range of vancomycin concentration (MIC between 4 to ≥ 1,024 μg/ml) and are susceptible to teicoplanin. *van*D-type strains are resistant to moderate levels of vancomycin (MIC 128 μg/mL) and susceptible to teicoplanin, while *van*C, *van*E, and *van*G-type strains exhibit low-level resistance to vancomycin [3,9]. High level gentamicin resistance (HLGR) phenotype is due to the expression of bifunctional aminoglycoside-modifying enzymes [AAC(6')-APH(2")] that are encoded by the *aac*A-*aph*D gene (Gentamicin MICs range, ≥ 100–500 μg/ml) [10,11].

Enterococcal infections are treated with ampicillin only or with a combination of ampicillin and aminoglycoside to achieve synergistic bactericidal activity. However, for patients who are allergic to penicillin or are infected by bacteria with high resistance to ampicillin and aminoglycosides, vancomycin is the powerful, last resort and alternative drug of choice. Intrinsic low-level resistance to aminoglycosides is an inherent property of enterococci. However, the prevalence of high level gentamicin resistance in enterococci is predictive of a loss of synergy between gentamicin and cell wall active agent such as ampicillin or vancomycin [3].

Nucleic acid-based tests using PCR are increasingly being used in laboratories to replace time-consuming, labor-intensive and less sensitive conventional diagnostic methods such as biochemical identification and Kirby-Bauer antimicrobial susceptibility tests. Various PCR methods have been developed to identify the (i) *Enterococcus *genus [12], (ii) vancomycin resistance [13], and (iii) high level aminoglycoside resistance [14]. The above methods do not detect all of the above mentioned targets simultaneously. Hence, the present study was focused to design a nanoplex PCR of vancomycin and bifunctional aminoglycoside resistant enterococci (V-BiA-RE) with an internal control for the detection of (i) *Enterococcus *genus and clinically important *Enterococcus *spp., namely *E. faecalis *and *E. faecium*, (ii) most common vancomycin resistant genotypes namely *van*A, B, C, D and (iii) high level gentamicin resistance. In order to rule out false negative results, an internal control was also included in the nanoplex PCR assay.

## Results

In the present study the V-BiA-RE nanoplex PCR was optimized successfully to identify 8 genes of *Enterococcus *genus, species (*E. faecalis *and *E. faecium*), vancomycin resistance (*van*A, *van*B, *van*C and *van*D) and high level gentamicin resistance (*aac*A-*aph*D) simultaneously. Stepwise optimization of primer concentration, annealing temperature, MgCl_2_, dNTP and Taq polymerase was done. The V-BiA-RE nanoplex PCR gave the best results when 4 mM MgCl_2_, 300 μM dNTP, 2 U Taq polymerase and 65°C annealing temperature was used.

The analytical sensitivity of the nanoplex PCR at the DNA level was found to be 1 ng of DNA (data not shown), whereas at the bacterial level it was found to be 10^5 ^CFU/mL (data not shown).

**Figure 1:**
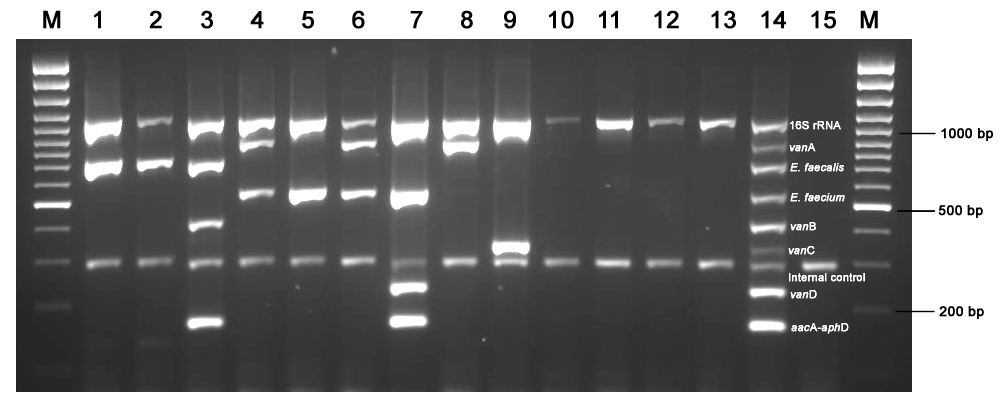
V-BiA-RE nanoplex PCR assay profile with reference strains. M, 100 bp plus marker; Lane 1, LMG 17122 (16S rRNA and* ddl*-*E. faecalis*); lane 2, LMG 8222 (16S rRNA and *ddl*-*E. faecalis*); lane 3, LMG 16216 (16S rRNA, *ddl*-*E. faecalis, vanB *and *aac*A-*aph*D); lane 4, LMG 16004 (16S rRNA, *ddl*-*E. faecium *and *van*A); lane 5, LMG 16200 (16S rRNA and *ddl*-*E. faecium*); lane 6, LMG 16192 (16S rRNA, *ddl*-*E. faecium *and* van*A); lane 7, BM 4339 (16S rRNA, *ddl*-*E. faecium*, *van*D and *aac*A-*aph*D); lane 8, LMG 16172 *E. durans *(16S rRNA and *van*A); lane 9, *E. casseliflavus *(16S rRNA and *van*C); lane 10, LMG 12172 *E. raffinosus *(16S rRNA); lane 11, LMG 12308 *E. mundti *(16S rRNA); lane 12, LMG 6399 *E. hirae *(16S rRNA); lane 13, LMG 10744 *E. avium *(16S rRNA); lane 14, positive control and lane 15, negative control.

The analytical specificity of the nanoplex PCR assay at the genus level was determined using 13 enterococcal reference strains and found to be positive for the *Enterococcus *genus specific 16S rRNA gene. A representative gel picture of V-BiA-RE with reference strains is shown in Figure 1, while the other 10 Gram-positive and 20 Gram-negative strains were negative. At the species level, all the reference strains of *E. faecalis *and *E. faecium *were positive for *ddl *gene by nanoplex PCR, while other *Enterococcus *species were negative (Table 1). The vancomycin and gentamicin resistant reference strains were positive for *van*A, *van*B, *van*C, *van*D or *aac*A-*aph*D genes by nanoplex PCR. However, the vancomycin and gentamicin sensitive reference strains were negative for *van*A, *van*B, *van*C, *van*D or *aac*A-*aph*D genes by nanoplex PCR (Table 1). Overall, the analytical specificity of nanoplex PCR was 100% for the detection of vancomycin and gentamicin resistant reference strains.

**Table 1:** Bacterial species and strains used in this study and results of V-BiA-RE nanoplex PCR.

**No.**	**Reference strains**	**16S rRNA**^a^	***ddl-E. faecalis***	***ddl-E. faecium***	***van*A**	***van*B**	***van*C**	***vanD***	***aac*A-*aph*D**^b^	**Internal control**
1.	*E. faecium *LMG 16004^c^	+	-	+	+	-	-	-	-	+
2.	*E. durans *LMG 16172^c^	+	-	-	+	-	-	-	-	+
3.	*E. faecium *LMG 16192^c^	+	-	+	+	-	-	-	-	+
4.	*E. faecalis *LMG 8222^c^	+	+	-	-	-	-	-	-	+
5.	*E. faecalis *LMG 17122^c^	+	+	-	-	-	-	-	-	+
6.	*E. faecalis *LMG 16216^c^	+	+	-	-	+	-	-	+	+
7.	*E. faecium *LMG 16200^c^	+	-	+	-	-	-	-	-	+
8.	*E. raffinosus *LMG 12172^c^	+	-	-	-	-	-	-	-	+
9.	*E. mundti *LMG 12308^c^	+	-	-	-	-	-	-	-	+
10.	*E. hirae *LMG 6399^c^	+	-	-	-	-	-	-	-	+
11.	*E. avium*, LMG 10744^c^	+	-	-	-	-	-	-	-	+
12.	*E. faecium *BM 4339 ^d^	+	-	+	-	-	-	+	+	+
13.	*E. casseliflavus*^e^	+	-	-	-	-	+	-	-	+
14.	*S. aureus*^f^	-	-	-	-	-	-	-	-	+
15.	*Streptococcus *spp. Group A^f^	-	-	-	-	-	-	-	-	+
16.	*Streptococcus *spp. Group B^f^	-	-	-	-	-	-	-	-	+
17.	*Streptococcus *spp. Group G^f^	-	-	-	-	-	-	-	-	+
18.	*Streptococcus *spp. Group F^f^	-	-	-	-	-	-	-	-	+
19.	*Bacillus *spp.^f^	-	-	-	-	-	-	-	-	+
20.	*Listeria *spp.^f^	-	-	-	-	-	-	-	-	+
21.	*Corynebacterium *spp.^f^	-	-	-	-	-	-	-	-	+
22.	*Gardnerella *spp.^f^	-	-	-	-	-	-	-	-	+
23.	*Lactobacillus *spp.^f^	-	-	-	-	-	-	-	-	+
24.	*E. coli *(EHEC)^f^	-	-	-	-	+	-	-	-	+
25.	*E. coli *(EPEC)^f^	-	-	-	-	-	-	-	-	+
26.	*E. coli *(ETEC)^f^	-	-	-	-	+	-	-	-	+
27.	*V. cholerae *(O1 classical)^f^	-	-	-	-	-	-	-	-	+
28.	*V. cholerae *O139 ^f^	-	-	-	-	-	-	-	-	+
29.	*V. mimicus*^f^	-	-	-	-	-	-	-	-	+
30.	*V. cincinnatiensis*^f^	-	-	-	-	-	-	-	-	+
31.	*V. furnissii*^f^	-	-	-	-	-	-	-	-	+
32.	*V. parahaemolyticus*^f^	-	-	-	-	-	-	-	-	+
33.	*S. typhi*^f^	-	-	-	-	-	-	-	-	+
34.	*S. sonnei*^f^	-	-	-	-	-	-	-	-	+
35.	*S. enterica*^f^	-	-	-	-	-	-	-	-	+
36.	*S. dysenteriae*^f^	-	-	-	-	-	-	-	-	+
37.	*S. boydii*^f^	-	-	-	-	-	-	-	-	+
38.	*C. freundii*^f^	-	-	-	-	-	-	-	-	+
39.	*Y. enterocolitica*^f^	-	-	-	-	-	-	-	-	+
40.	*P. mirabilis*^f^	-	-	-	-	-	-	-	-	+
41.	*P. aeruginosa*^f^	-	-	-	-	-	-	-	-	+
42.	*K. pneumoniae*^f^	-	-	-	-	-	-	-	-	+
43.	*P. Shigelloides*^f^	-	-	-	-	-	-	-	-	+

^a^*Enterococcus *genus

^b ^Bifunctional aminoglycoside resistant genotype

^c ^Reference strains from Belgian Co-ordinated Collections of Micro-organisms (BCCM), Ghent, Belgium

^d ^Kindly provided by Professor. Patrice Courvalin and Dr. Bruno Perichon, Institut Pasteur, Paris, France

^e ^Obtained from Institute for Medical Research, Malaysia

^f ^Department of Medical Microbiology and Parasitology, School of Medical Sciences, Universiti Sains Malaysia. '+' is positive; '-' is negative by V-BiA-RE PCR

The DNA sequencing results of the PCR amplicon for the 8 genes were contig aligned using the ContigExpress aligment program and were analyzed by BLAST. The results showed that all 8 PCR amplicons were specific to their respective genes and had 91–100% sequence identity with the existing Genbank sequences.

Upon completion of the standardization of the V-BiA-RE nanoplex PCR with reference strains, the assay was validated with 159 clinical isolates. Among the 159 clinical isolates 154 (97%) were found to be *E. faecalis *and only 5 (3%) were *E. faecium*. Of the five *E. faecium *isolates, four were from blood samples and one was from urine sample. Of the remaining 154 *E. faecalis *isolates, 34 were from blood samples, 59 from urine samples, 42 from pus samples, 6 from high vaginal swab samples, 10 from body fluid samples, 1 from a peritoneum dialysis fluid sample and 2 from urine catheter samples. Thus, the clinical isolates had a high percentage of *E. faecalis *infections. However, none of the clinical *Enterococcus *isolates showed vancomycin resistance.

Among the 159 enterococci isolates, 42 had *aac*A-*aph*D gene by V-BiA-Re nanoplex PCR. Of the 42 isolates, 39 were *E. faecalis *while 3 were *E. faecium*. However, by the conventional antimicrobial susceptibility method 41 of them were HLGR enterococci (Gentamicin MIC ≥ 100–500 μg/mL). One of the *E. faecium *isolate that was positive for *aac*A-*aph*D gene by nanoplex PCR was found to be sensitive to gentamicin by conventional MIC method. Though genotypically the *aac*A-*aph*D gene was detected and confirmed by PCR and sequencing, it is possible that the *aac*A-*aph*D gene is non-functional and is not expressed phenotypically or due to the presence of pseudogene [15].

The diagnostic accuracy of V-BiA-RE nanoplex PCR at the genus and species level was determined using 159 clinical isolates and found to have 100% sensitivity, specificity, positive and negative predictive values. However, the V-BiA-RE nanoplex PCR for the *aac*A-*aph*D gene detection showed 97.7% sensitivity, 100% specificity, 100% positive and 99.4% negative predictive values in detecting HLGR enterococci.

## Discussion

The present study is unique since it is the first study where we have developed a combined molecular test for the determination of the *Enterococcus *genus, two clinically important species (*E. faecalis *and *E. faecium*) and *van*A-D genotypes and high level gentamicin resistance (HLGR) simultaneously. Currently the available assay is able to detect either gentamicin or vancomycin resistance [10-13]. Though there are numerous reports on PCR assays for the detection of VREs [13,16], only few of them have incorporated internal controls in their assays to rule out false negatives [17,18]. According to CLSI guidelines for Molecular Diagnostic Methods for Infectious Diseases (MM3-A2), incorporation of an internal control in the reaction is essential for the diagnostic test to exclude false negative result or the presence of inhibitors [19]. In the present study, the inclusion of the 300 bp internal control in the V-BiA-RE nanoplex PCR assay helped us to rule out false negatives or PCR inhibitors.

The V-BiA-RE nanoplex PCR was found to be 100% sensitive and specific in detecting known vancomycin and gentamicin resistance genes in Enterococcal strains. The use of V-BiA-RE PCR as a diagnostic tool was also studied by analyzing 159 clinical isolates obtained from 3 different hospitals in Malaysia. We found that most of them were *E. faecalis *(94%) and all of them were susceptible to vancomycin. However, 26% were resistant to HLGR. These results are consistent with results of other groups, where they have shown that *E. faecalis *is predominant among the clinical isolates and were vancomycin sensitive though some were HLGR enterococci [3,14].

In Malaysia the presence of VREs has been reported from clinical [5-7] and farm animal samples [16,20]. Sporadic cases of VRE infection and colonization have also been reported in the neighboring country, Singapore [4]. Since antimicrobial-resistant enterococci have been detected in livestock in Malaysia, there could be a possible epidemiological link between livestock origin and human infections [4-7,16,20]. Hence, characterization of VRE isolates from human stools and livestock animals is needed, especially in areas where antibiotics can be obtained from any drug store or over-the-counter and where farmers feed their chickens with antibiotics to promote growth [3,15].

The percentage of HLGR enterococci observed in this study (26%) is relatively lower than those reported in China (64%) [10] and Greece (42%) [11]. In both studies, the HLGR enterococci were mostly associated with the presence of *aac*A-*aph*D gene as observed in this study.

Genomic DNA extracted from two of the *E. coli *(EHEC and ETEC) strains found to have *van*B gene like products (Table 1). It has been documented that the *E. coli *possess homologous genes called *ddl*A and *ddl*B [GenBank accession: U00096] that have 29–38% amino acid identity to *van*A and *van*C genes [21]. However sequencing of these PCR product revealed that it is similar to a hypothetical protein present in *E. coli *(Gene-Ecs5269, GenBank accession number BA000007) and is not related to *ddl*A/*ddl*B genes of *E. coli*. It is possible that in the nanoplex PCR assay, the presence of 9 pairs of primers might have lead to non-specific PCR product. These types of false positive results do not arise when Enterococci broth is used as an enrichment medium, since this medium is known to inhibit Gram-negative bacterial (*E. coli*) growth.

The limitations of the conventional antimicrobial susceptibility tests are that the susceptibility pattern varies with inoculum size. In addition, there is no standardized MIC cut-off value available for *van*A, *van*B, *van*C1/C2/C3 and *van*D in conventional antimicrobial susceptibility tests. Currently, it is not a usual practice in laboratories to identify the species of *Enterococcus*, except in cases of septicemia. Genotype-based molecular tests can overcome the limitation, where the presence of the gene is accurately detected when the CFUs of the bacteria are more than the analytical sensitivity level. Moreover, rapid detection of antibiotic resistant bacteria is important not only for therapeutic decisions, but also for infection control [15].

The *van*E and *van*G primers were not included in the V-BiA-RE nanoplex PCR since these genotypes are not common among *Enterococcus *spp. [22]. The V-BiA-RE nanoplex PCR assay developed in the present study will be useful in the epidemiological screening of VRE carriers or reservoirs. We are currently evaluating this assay for screening VRE carriage in human stools and poultry farms in Malaysia.

## Conclusion

Bacterial lysates prepared by a simple boiling method without lysis buffer and lytic enzymes were used as templates in the V-BiA-RE nanoplex PCR assay. The PCR assay was able to detect 8 genes that are essential for the identification of the most common enterococci spp. and their vancomycin and gentamicin resistance genotypes simultaneously in a single test within 4 hours. The built-in internal control in this assay prevents the false negative cases. The diagnostic accuracy was determined using the 159 clinical specimens which showed 97% of the clinical isolates belonged to *E. faecalis*, of which 26% showed HLGR genotype, but none were vancomycin resistant. Hence, this test can be used as an effective diagnostic and surveillance tool to monitor the spread and emergence of VRE.

## Methods

### Study design

This is a retrospective diagnostic study where the sample size was calculated by single proportion test based on the prevalence of 12.3% [23] and 95% Confidence Interval with expected specificity of 92%. The study was approved by the Research and Ethics Committee, School of Medical Sciences, Universiti Sains Malaysia.

### Bacterial strains and clinical specimens

The reference *Enterococcus *spp. and other bacteria used in this study are listed in Table 1. A total of 159 *Enterococcus *spp. isolated from routine clinical specimens obtained from three hospitals namely, Hospital Universiti Sains Malaysia (HUSM), General Hospital Kota Bharu (GHKB) and Hospital Tengku Ampuan Afzan, Kuantan, Malaysia from May to October 2004 were used in this study. Of these 159 isolates, 38 were isolated from blood samples, 60 from urine samples, 42 from pus samples, 6 from high vaginal swabs, 10 from body fluid samples, 1 from peritoneum dialysis fluid and 2 from urine catheter samples.

### Screening of *Enterococcus* spp. from clinical specimens by conventional method

The clinical isolates were inoculated into Enterococci broth (Merck, Darmstadt, Germany) and Enterococcosel agar [Becton Dickinson (BD), BBL, New Jersey, USA] without the addition of vancomycin and incubated at 37°C for 24 hours. The isolates that showed growth in both media were selected for biochemical identification and antimicrobial susceptibility testing.

To differentiate *Enterococcus *from other Group D streptococci, isolated colonies from clinical specimens were tested by standard biochemical tests (PYR: Oxoid, United Kingdom, England; Enterococcosel agar: BD, BBL, New Jersey, USA and 6.5% NaCl brain heart infusion (BHI) broth: Oxoid, United Kingdom, England). Further confirmation to the species level was carried out as described by Facklam and Waitkins [24,25]. The reference strains *E. raffinosus *(LMG 12172) and *E. durans *(LMG16172) (Belgian Co-ordinated Collections of Micro-organisms, BCCM, Ghent, Belgium) were used as controls for the above biochemical tests.

Minimum inhibitory concentration (MIC) of vancomycin (CheilJedang, Korea), teicoplanin (Gruppo Lepetit, Italy) and gentamicin (Duopharma, Malaysia) was determined by agar dilution method according to CLSI (Clinical and Laboratory Standards Institute) guidelines with slight modification based on the procedures as described by Hayes *et al.*[26]. Furthermore, E-test kits (AB Biodisk, Solna, Sweden) were also used to determine the susceptibility patterns of vancomycin. The results were categorized according to CLSI standards. Reference strains used as controls were *E. faecium *(LMG 16192), *E. faecalis *(LMG 16216), *E. faecium *(BM 4339) and *E. casseliflavus *(Table 1).

### Primer designing for V-BiA-RE nanoplex PCR

The 16S rRNA of *Enterococcus *genus, *ddl *of *E. faecium*, *ddl *of *E. faecalis, van*A, *van*B, *van*C, *van*D, and *aac*A-*aph*D gene sequences were obtained from GenBank [27] for DNA sequence alignment and primer design. The ClustalW program in Vector NTI version 9.0 software (Invitrogen Corporation, California, USA) was used to align the DNA sequences. The conserved and non-conserved regions of the DNA sequence alignments were visualized using GeneDoc software [28]. Based on the conserved regions of the alignment, specific primer pairs were designed to amplify the *Enterococcus *genus. Specific primers of *E. faecium *and *E. faecalis *species were designed based on the non-conserved regions of *ddl *gene sequences. Vancomycin resistance specific primers were designed based on the non-conserved regions of *van*A, *van*B, *van*C, and *van*D DNA sequences. High level gentamicin resistance (HLGR) specific primers were designed based on *aac*A-*aph*D gene. The 8 primer pairs were designed in such a way that the PCR products ranged from 150 bp to 1200 bp. The specificity of the designed primers was checked using BLAST available at the GenBank website [29]. The primer sequences for the 8 genes and expected PCR product sizes are shown in Table 2. A primer pair based on *ctx*A gene was designed and was used as an internal control.

**Table 2:** Sequences of primers used for the V-BiA-RE nanoplex PCR.

**Gene**	**Primer Name**	**5'--------------------------------3'**	**GenBank Accession Number**	**Product Size**
**16S rRNA**	16SrRNA-F	AGG GGA TAA CAC TTG GAA ACA	AB015233	1178 bp
	16SrRNA-R	TTC GCG ACT CGT TG TAC TTC		
***ddl E. faecalis***	Elis-F2	GGC CCT CTT TTA TCT GAA CGA	U00457	734 bp
	Elis-R3	GCG ACT TAA GCC ACT TCC AT		
***ddl E. faecium***	Ecium-F	CGC AGA GCA TGA AGT GTC CA	AF550665	557 bp
	Ecium-R2	CTT CTC GGT TTT CTG CTT TTG TA		
***Van*A**	vanA-F	TTG GGG GTT GCT CAG AGG AG	X56895	931 bp
	vanA-R	CTT CGT TCA GTA CAA TGC GG		
***Van*B**	vanB-F	AAT GCG GGG AGG ATG GTG CG	AF550667	446 bp
	vanB-R	GAT GCG GAA GAT ACC GTG GC		
***Van*C**	vanC-F2	GCA GGT TCT GCC TTA TGT ATG AA	AF162694, AY033764, L29638	339 bp
	vanC-R	ATG AAA TGG CGT CAC AAG CA		
***Van*D**	vanD-F	CGT ATG TGG GAT GCG ATA TTC AA	AF130997	230 bp
	vanD-R2	CTT CGA TTG CTG CCT GCA GTT		
***aac*A-*aph*D**	acph-F1L	GAT TTG CCA GAA CAT GAA TTA CAC GA	AY602207	156 bp
	acph-R1L	CAT AAC CAC TAC CGA TTA TTT CAA T		
**Internal Control (*ctx*A)**	IC-F	AAC TCA GAC GGG ATT TGT TAG GC	AF510996	300 bp
	IC-R	TCT CTG TAG CCC CTA TTA CGA TGT		

### **V-BiA-RE nanoplex PCR assay**

The monoplex PCR for each gene and the V-BiA-RE nanoplex PCR assays were standardized using genomic DNA extracted from reference *Enterococcus *spp. A mixture of DNAs from 4 reference strains namely *E. faecium *(LMG 16192), *E. faecalis *(LMG 16216), *E. faecium *(BM 4339) and *E. casseliflavus *that contained the 8 genes of interest was used as a positive control. DNase-free distilled water was used as a negative control. In addition, a plasmid containing *ctx*A gene (1 pg) was used as a template for the internal control. To rule out false negative activities, an internal control (primers pair and template) was incorporated in every reaction mixture including negative controls.

The diagnostic evaluation of the nanoplex PCR was done using the lysates from 159 clinical isolates. The isolated colonies from Enterococcosel agar were inoculated into LB broth and incubated at 37°C for 24 hours. Bacterial lysates for PCR were prepared by centrifuging the 100 μl of culture at 10,000 × *g *for 3 minutes, the supernatant were removed and the pellets were resuspended in 100 μl of DNase-free distilled water. The suspensions were boiled in a water bath for 10 minutes and centrifuged again at 10,000 × *g *for 3 minutes. Then, 2 μl of the supernatants (lysates) were used in the V-BiA-RE nanoplex PCR assays.

The optimized concentration of primer for each gene (0.2 pmol 16S rRNA, 0.8 pmol *ddl-E. faecium*, 0.8 pmol *ddl-E. faecalis*, 0.8 pmol *van*A, 0.05 pmol *van*B, 0.7 pmol *van*C, 0.4 pmol *van*D, 1 pmol *aac*A-*aph*D and 0.2 pmol *ctx*A) was used in the V-BiA-RE nanoplex PCR. The other components used in the PCR were 300 μM dNTPs, 4 mM MgCl_2_, 1× PCR buffer and 2 U *Taq *DNA polymerase (Fermentas, Vilnius, Lithuania). The PCR was carried out using a Mastercycler Gradient (Eppendorf, Hamburg, Germany) with one cycle of initial denaturation at 95°C for 5 min, 30 cycles of denaturation at 95°C for 30 sec, annealing for 30 sec at 65°C and extension at 72°C for 30 sec, followed by an extra cycle of annealing at 65°C for 30 sec and a final extension at 72°C for 5 min. The PCR products were analyzed by electrophoresis on 2% low EEO agarose gels (Promega, Madison, USA) with ethidium bromide at 90 Volts for 75–90 min. PCR products were visualized under UV illumination and photographed using an image analyzer (ChemiImager 5500, Alpha Innotech, California, USA).

### Confirmation of the PCR amplicons by DNA sequencing

All eight PCR products of the different genes that were obtained from reference strains (Table 1) were cloned into a PCR cloning vector pTZ57T/R (Fermentas, Vilnius, Lithuania) and sequenced using an automated DNA sequencer at Tech Dragon Ltd (Hong Kong, China). The DNA sequencing results were analyzed using the ContigExpress alignment program in Vector NTI version 9.0 software (Invitrogen corporation, California, USA). Resultant contigs were analyzed by BLAST [29] provided by the National Center for Biotechnology Information to calculate the percentage identity.

### Evaluation of V-BiA-RE nanoplex PCR assay

Analytical specificity was evaluated using DNA lysates prepared from pure cultures of 13 phenotypically and genotypically well-characterized *Enterococcus *spp. and 10 Gram-positive and 20 Gram-negative strains obtained from different sources (Table 1).

The analytical sensitivity was evaluated using various concentrations of genomic DNA starting from 1 μg to 10 pg and lysate starting from 10^8 ^– 10^3 ^CFU/ml obtained from a reference strain, *E. faecalis *(LMG 16216).

The diagnostic evaluation of the nanoplex PCR was carried out using 159 clinical isolates. The results were compared with the conventional microbiological, biochemical, and antimicrobial susceptibility tests which were considered as the 'gold standard' [30].

### Statistical analysis

The clinical sensitivity, specificity, positive (PV^+^) and negative predictive value (PV^-^) of V-BiA-RE nanoplex PCR were calculated based on the CLSI Guidelines for Molecular Diagnostic Methods for Infectious Diseases [30].

## Authors' contributions

CYY carried out the DNA sequence alignment, designed the primers, developed multiplex PCR, analyzed clinical samples and helped to draft the manuscript. LSY contributed in the multiplex PCR optimization, sample analysis and was involved in drafting of the manuscript. PL participated in the study design, primer design, data analysis and critically edited and revised the manuscript. MR conceived and coordinated the study, helped in DNA sequence analysis and primer design, data analysis and drafted the manuscript. All authors read and approved the final manuscript.
